# The social aspects of genome editing: publics as stakeholders, populations and participants in animal research

**DOI:** 10.1177/0023677221993157

**Published:** 2021-02-17

**Authors:** Gail Davies, Richard Gorman, Renelle McGlacken, Sara Peres

**Affiliations:** 1 Geography Department, University of Exeter, UK; 2 School of Sociology and Social Policy, University of Nottingham, UK; 3 School of Geography and Environmental Sciences, University of Southampton, UK

**Keywords:** public opinion, public engagement, patient involvement, genome editing, animal research

## Abstract

The application of genome editing to animal research connects to a wide variety of policy concerns and public conversations. We suggest focusing narrowly on public opinion of genome editing is to overlook the range of positions from which people are brought into relationships with animal research through these technologies. In this paper, we explore three key roles that publics are playing in the development of genome editing techniques applied to animals in biomedical research. First, publics are positioned by surveys and focus groups as stakeholders with opinions that matter to the development of research technologies. Learning lessons from controversies over genetically modified food in Europe, these methods are used to identify problems in science–society relations that need to be managed. Second, people are recruited into research projects through participating in biobanks and providing data, where their contributions are encouraged by appeals to the public good and maintained by public confidence. Thirdly, patients are increasingly taking positions within research governance, as lay reviewers on funding panels, where their expertise helps align research priorities and practices with public expectations of research. These plural publics do not easily aggregate into a simple or singular public opinion on genome editing. We conclude by suggesting more attention is needed to the multiple roles that different publics expect – and are expected – to play in the future development of genomic technologies.

## Résumé

L'application de l'édition génomique à la recherche sur les animaux est liée à une grande variété de préoccupations stratégiques et de conversations publiques. Nous suggérons que se concentrer étroitement sur l'opinion publique de l'édition génomique revient à négliger l'éventail de positions à partir desquelles les gens sont amenés à la recherche animale par le biais de ces technologies. Dans cet article, nous explorons trois rôles clés que jouent les publics dans le développement des techniques d'édition génomique appliquées aux animaux utilisés dans la recherche biomédicale. Premièrement, les publics sont positionnés par des enquêtes et des groupes de discussion comme des intervenants ayant des opinions importantes pour le développement des technologies de recherche. En tirant des leçons des controverses que suscitent les aliments génétiquement modifiés en Europe, ces méthodes sont utilisées pour identifier les problèmes liés aux relations science-société qui doivent être gérés. Deuxièmement, les personnes sont recrutées dans des projets de recherche en participant à des biobanques et en fournissant des données, où leurs contributions sont encouragées par des appels au bien public et maintenues par la confiance du public. Troisièmement, les patients prennent de plus en plus de positions dans le domaine de la gouvernance de la recherche, en tant qu'examinateurs externes de comités de financement, où leur expertise aide à aligner les priorités et les pratiques de recherche sur les attentes du public à l'égard de la recherche. Ces publics pluriels ne se regroupent pas facilement en une seule opinion publique simple ou singulière sur l'édition génomique. Nous concluons en suggérant qu’il est nécessaire d’accorder plus d’attention aux rôles multiples que les différents publics s’attendent à jouer – et à ceux que l’on s’attend à ce qu’ils jouent – dans le développement futur des technologies génomiques.

## Abstract (de)

Zur Anwendung der Genom-Editierung in der Tierforschung gibt es viele politische Bedenken und öffentliche Debatten. Wir geben hier zu bedenken, dass bei einer einseitigen Berücksichtigung der öffentlichen Meinung zur Genom-Editierung eine ganze Bandbreite an Positionen außer Acht bleibt, auf deren Grundlage Menschen durch diese Technologien einen Bezug zur Tierforschung erlangen. In diesem Beitrag untersuchen wir drei Schlüsselrollen, die die Öffentlichkeit bei der Entwicklung von Genome-Editing-Techniken spielt, die in der biomedizinischen Forschung bei Tieren zum Einsatz kommen. Erstens positionieren Umfragen und Fokusgruppen die Öffentlichkeit als Beteiligte mit Meinungen, die für die Entwicklung von Forschungstechnologien von Bedeutung sind. Unter Nutzung der aus den Kontroversen um gentechnisch veränderte Lebensmittel in Europa gezogenen Lehren werden diese Methoden genutzt, um Probleme in den Beziehungen zwischen Wissenschaft und Gesellschaft zu identifizieren, die es zu bewältigen gilt. Zweitens werden Menschen für Forschungsprojekte rekrutiert, um an Biobanken teilzunehmen und Daten zur Verfügung zu stellen, wobei ihre Mitwirkung durch Appelle an das Gemeinwohl gefördert und durch öffentliches Vertrauen gewährleistet wird. Drittens nehmen Patienten zunehmend Positionen in der Governance-Forschung ein, z. B. als Laiengutachter in Finanzierungsgremien, wo ihr Fachwissen dazu beiträgt, Forschungsprioritäten und -praktiken mit den öffentlichen Erwartungen an die Forschung in Einklang zu bringen. Diese Bandbreite öffentlicher Meinung lässt sich nicht ohne weiteres zu einer einfachen oder singulären öffentlichen Meinung über Genome Editing zusammenfassen. Wir ziehen daher abschließend das Fazit, dass den vielfältigen Rollen, die verschiedene Teile der Öffentlichkeit bei der zukünftigen Entwicklung von Genomverfahren erwarten – und von denen erwartet wird, dass sie sie wahrnehmen – mehr Aufmerksamkeit zuteil werden muss.

## Resumen

La aplicación de la edición genómica en la investigación animal conecta con una amplia variedad de temas de políticas y conversaciones públicas. Pensamos que centrarse limitadamente en la opinión pública de la edición genómica es ignorar la gama de puestos desde los que la gente entra en contacto con la investigación animal a través de estas tecnologías. En este estudio, exploramos tres funciones principales que el público está desempeñando en el desarrollo de técnicas de edición genómica aplicadas a animales en la investigación biomédica. En primer lugar, el público se posiciona mediante encuestas y grupos de debate como actores con opiniones que son importantes para el desarrollo de tecnologías de investigación. Tras aprender de distintas controversias sobre alimentos modificados genéticamente en Europa, estos métodos se utilizan para identificar problemas en las relaciones de la ciencia con la sociedad que tienen que gestionarse. En segundo lugar, la gente es reclutada para proyectos de investigación mediante la participación en biobancos y suministrando datos, donde se fomenta su contribución mediante llamadas al bien público y se mantiene con la confianza del público. En tercer lugar, los pacientes cada vez están consiguiendo puestos dentro de la gobernanza de la investigación, como revisores de colocaciones en paneles de financiación, donde su pericia ayuda a alinear prioridades y prácticas de investigación con las expectativas que el público tiene en la investigación. Este público plural no se conjuga fácilmente en una opinión pública simple o singular sobre la edición genómica. Concluimos este estudio sugiriendo una mayor atención en las múltiples funciones que diferentes públicos esperan desempeñar (y que se espera que lo hagan) en el futuro desarrollo de tecnologías genómicas.

## Introduction

The application of genome editing to animal research connects a surprisingly diverse set of policy concerns and public conversations. Initial plans to do research using genome editing techniques could be reviewed by a patient volunteer sitting on a funding panel, who is attuned to the public priorities and purpose of the research. The project licence application will be reviewed by a local animal welfare and ethical review board, where there is likely to be a lay representative speaking about social perspectives on acceptable animal use. The research itself may involve screening animal phenotypes through protocols devised by gathering patient experiences from surveys administered through clinical practice. The ultimate clinical development and implementation of therapeutic products will depend on recruitment of people into clinical trials to test safety and efficacy, but also on there being a wider publicly acceptable regulatory framework underpinning this use of animals in research. At each point there are different public interests and there will be different public views. The social aspects of applying genome editing to animal research are both distributed and diverse, meaning they are demanding for policy makers and practitioners to pull together.

The Royal Society^1^ and the Nuffield Council for Bioethics^2^ recently published their public dialogues on the social aspects of genome editing. These show the wide range of social concerns around genome editing and demonstrate how different methods for engaging people in dialogue shape how they talk and think about issues. This adds further methodological complexity to the diversity of roles people play. Social scientists have long argued that public opinion on new technologies does not reflect pre-existing individual attitudes; rather attitudes emerge from the way institutions and methods seek to engage people and their everyday experiences.^3,4^ Social science methods, policy dialogue and media campaigns all structure how public voices and interests are expressed and heard.^4,5^ Looking at how these publics take shape is important for devising methods that engage people in genuine dialogue with the emerging technologies affecting them.

In what follows we unpack three key roles that publics are playing in conversations about genome editing applied to animals in biomedicine: as stakeholders, populations and participants in research. In each case, we explore how different research techniques are used to recruit and represent these publics, focusing on surveys and public dialogue, biobank participation and patient involvement. We consider the continuities between past debates on genetic modification, which took place from the late 1990s and focused on gene patenting, transgenics and cloning,^6^ and the results of polls and public dialogue methods used to assess public views around genome editing today. We also trace the new ways people are involved in the interfaces between animal research and genome editing, through contributing data to biobanks and patient involvement. We draw on collaborative work within the Wellcome Trust funded Animal Research Nexus programme^7^ to map out these different public interfaces, focusing mainly on the UK. The multiple roles that publics play in the future development of genome technologies will differ internationally, but this complexity will be found everywhere in different configurations.

## Searching for the public

We start with the assertion ‘the public’ is not something pre-existing but is something that is called into being and always encountered in mediated forms.^8^ We do not dismiss terms like ‘public opinion’ or the ‘public interest’, but we do want to contextualise them. In liberal democracies, we recognise we are talking about something important when we talk about the public interest, identifying an issue that matters to everyone, requires collective decisions, and warrants public debate.^9^ The aspiration to record what the public thinks about animal research and genome editing reflects these important values. Yet to search for *the* public view on something is to pursue a mirage; it fades as we get closer to it. Members of the public may be asked their opinions through a variety of methods – such as surveys, interviews, or focus groups – carried out by a range of researchers – in academic institutions, market research, or policy contexts. That this gives rise to a diversity of views is generally accepted and the earlier literatures on genetic modification of animals indicate there are differences by gender, age, occupation and education.^10^ However, there are further differences that make aggregation hard. Members of the public may respond to surveys that position them as citizens. They may also contribute to the policy and practice of genome editing through engaging in consultations, sitting on ethics committees, participating in preclinical research, or campaigning in groups, speaking in different ways that reflect these particular contexts.

Using the plural term ‘publics’ recognises the demographic differences and divergent opinions around a controversial topic like animal research. It also incorporates the way people speak and act from different positions when they make their views public. You can see this through considering what social scientists call everyday ‘speech acts’.^11^ When asked their opinion, people often use phrases such ‘as a parent’, ‘as a local resident’, or ‘as a taxpayer’. Each is a different kind of public voice, located in different relationships and communities, with different expectations of the people and institutions they are speaking to. We may use terms in sequence to express ambiguity: ‘as a consumer, I do not want to pay more for the food I need to feed my family’, but ‘as a pet owner, animal welfare is important’. Through speaking, we are also acting, declaring what we care about, and what we want other people or authorities to do to align with our expectations – here provide affordable food, whilst protecting animal welfare. Personal actions follow similar patterns: we do and ask for different things when we understand ourselves as citizens, consumers, carers, or patients. We may also emphasise different aspects of these identities when research methods engage us as individuals (as in questionnaire surveys or polls) or as part of social groups (as in focus groups or committees). Advertisers, campaigners and politicians appeal to these different facets of our lives, giving rise to stratified, contradictory, and sometimes polarised public groups, or publics.

Understanding these diverse publics is vitally important to the unfolding politics and practice of genome editing. The development of genetic engineering – from transgenics to CRISPR/Cas technologies – has been accompanied by social research techniques for engaging publics in dialogue with its changing aspirations and implications.^12–14^ The Eurobarometer surveys have been polling publics on scientific issues since the 1990s,^15^ with a Special Eurobarometer on the genetic alteration of animals in 2010.^16^ These methods are used to measure opinion, monitor long-term trends and identify problems in science–society relations so that they may be resolved. The 2014 UK’s Ipsos MORI finding that the bioscience sector was seen as secretive and untrusted^17^ led to the Concordat on Openness in Animal Research, with signatories committing to providing ‘clear, transparent and open communication and proactive public engagement on this subject’.^18^

Regular polls on animal research have also been a significant resource for regulators, scientists and campaigning groups. These opinion polls indicate the continual conditionality of public support for animal research, where there are no alternatives and research is essential, and the variations in public trust and optimism over time.^17,19^ As Hobson-West explains, opinion polls are valued for making public views around this contentious issue tractable and conferring moral legitimacy and democratically accountability to those looking to evidence public views on animal research.^20^ Polls are important means of constructing the public view of both genome editing and animal research, but they are framed by institutional questions and present a partial picture of what matters to people. They can be supplemented by qualitative research showing how people talk about the genetic modification of animals, and what they are asking of others, in social contexts and conversations.

## Finding common concerns

Smith and Samuel currently suggest ‘non-human Genome Editing is a “technical category” but not a “public topic”’.^21^ They emphasise that ongoing expert discussions about technical and policy issues are not yet matched by public debate about genome editing and animals. Yet there are periods when the genetic alteration of animals has become a public topic from the first genetically modified organisms (GMOs) in 1973 to the present. These cycles of public interest follow scientific developments, non-governmental organisation campaigns, legal challenges and policy engagements. They often focus on charismatic animals, which capture media attention, or contentious issues, which fit existing media scripts about scientific intervention and control. The patenting of OncoMouse,^22^ the cloning of Dolly the sheep,^23^ the stops and starts of xenotransplantation,^24,25^ the regulatory challenge of hybrid embryos^26^ and the future potential of gene drives^27^ have all become public topics since the 1990s, accompanied by opinion polling and qualitative social research. Despite the changing focus of debates, these conversations share many characteristics as they are mediated through everyday experiences and media narratives.

In the early 2000s, Macnaghten^28^ ran focus groups exploring public attitudes and sensibilities towards animals and biotechnology in the UK. People’s concerns reflected how they valued animals in their everyday lives. They were worried genetic engineering would change animals ‘in their nature’, even unmooring their sense of the intrinsically ‘right’ way of being for animals. The use of animals in research was so far removed from everyday engagements with animals that many people did not want to think about it, but this did not indicate a lack of concern. All had their own understandings of care and responsibility, though there were differences between pet owners, wildlife enthusiasts, farmers, hunters and others. The genetic engineering of animals was more acceptable for medical uses than food, something also heard in debates about genetically modified plants, but the boundaries between the two were not seen as static or unalterable. The apparently contradictory values people have about different animals, derived from everyday experiences, is a common finding.

As people consider the ethical challenge of changing an animal’s genome, they also draw on a set of recurring vocabularies. Naturalness is a problematic category to define, but it is a recognised language through which people express ethical concerns.^6^ Phrases like ‘playing god’ or ‘going against nature’ are often dismissed as emotive or empty rhetoric,^29^ but a detailed look at these speech acts shows they are personally meaningful moral expressions and communicate views about institutional responsibility.^25^ People draw on common cultural images to raise questions about scientific and regulatory control. Frankenstein’s monster often accompanies genetic debates and is used by publics, campaigning groups and others to raise issues around accountability.^30^ Recent work on genome editing suggests similar issues of naturalness, trust and purpose do arise, though they also indicate potential differences by pointing to the greater precision and lack of species crossing in genome editing,^6^ something stressed to secure public consent.

Whilst many people resist thinking about animals as means to an end, the proposed purpose of genetic alteration is an enduring aspect of public concern. Studies since the 1990s repeatedly present evidence that the genetic alteration of organisms is more acceptable for medical developments than for agricultural purposes,^12^ in secure laboratories than open environments,^31^ and for societal benefits than for commercial gain.^12^ Cloning was seen as particularly troubling as its end purpose was not clear.^15^ People also expect these debates to be held in public and excluding them, for reasons of confidentiality or security, diminishes trust in both scientific and regulatory control.^6^ Whilst opinion polls have provided institutionally useful summaries of public views on genetic engineering in the past, qualitative research has helped show how these views are formed in relation to everyday experiences and shifting institutional conduct. Many of these patterns can be seen in the publics emerging around genome editing today.

## The emergent publics of genome editing

### Publics as stakeholders

Public engagement around genome editing is now seen as a priority for policy makers.^2,32^ Questions on the subject increasingly appear in opinion polls, as in the 2019 instalment of the UK Government’s ‘Public Attitudes to Science’ survey.^33^ There are also a growing number of dialogue events examining how people talk about genome editing. So far, lay discussion ‘if it occurs, appears to be predominantly invited and in formal spaces’.^21^ One example is the 2017 Royal Society’s public dialogue, which consisted of three deliberative workshops and a national survey.^2^ The workshops sought to identify ‘the frames and contexts that moderate the public acceptability of developing UK research into genetic technologies’, whilst the survey aimed to provide ‘clarity on the applications that a majority of the public do or do not support, why and under what conditions’.^1^ This process indicates the complex role of the public here: it recognises the public stakes in genome editing, the importance of having debates in public, and the legitimacy conferred by measuring public opinion.

However, looking closely at how different aspects of this process were organised helps explain why these different versions of the public do not always align. The Royal Society survey excluded those with ‘a professional stake in genetic technologies (e.g. clinicians, academics and policy makers in the field)’.^1^ Members of animal rights and welfare groups have historically also been screened out of public opinion polls concerning animal research.^19^ Excluding those with explicit interests or knowledge creates a version of the public that is seen as statistically unbiased, but also largely uninformed. Perhaps not surprisingly many polls then identify the public as ‘deficient and misguided’^34^ in their understanding of science. As Wehling observes, screening can hamper successful public participation by suppressing ‘precisely those attributes which would enable civil society actors to make meaningful contributions’.^35^

With few everyday contexts to ground discussions of non-human genome editing at present, the exclusion of those with existing knowledge makes public opinion more tractable, but it attenuates debate. The Royal Society workshops aimed to recruit ‘10% of people who have a specific interest in the application of genetic technologies under discussion to ensure it was an inclusive process encompassing a wide range of views’.^1^ However, these are seen as interest groups, not the view of ‘the public’. By pluralising publics social scientists acknowledge the different positions on issues such as genome editing and call for methods that account for and engage with this diversity.

### Publics as populations

Ethical debates around genome editing in biomedicine, which focus on issues of public good and the solidarities that promote research participation,^36,37^ have had less visibility in discussions of animal research. Mulvihill et al. suggest genome editing does not produce substantively new ethical issues in medicine, but the explosive rate of new findings, unrealistic expectations of professions and publics, and additional commercial imperatives does.^36^ Genome editing brings new contact points between publics and animal research through translational genomics, tissue biobanks and data intensive science. These seem far removed from conventional public controversies around animal research, but these publics are central to realising the promise of genome editing. They also raise familiar questions about how potential harms are controlled and benefits distributed, and similarly depend on public trust and ideas of collective good.

More people are now taking part in large-scale projects that involve animal research, such as the 100,000 genomes project,^38^ or contributing to biobanks, such as the UK Biobank.^39^ These aim to create collections of populations or rare diseases by inviting individuals to donate biological samples and permit access to health records and other data. These may be used in biomedical research involving animals, such as improving animal models^40^ or combining data from human and animal studies.^41^ This is a less visible but more direct form of public engagement with science than contributing to a public dialogue. It also depends on a new notion of the public as ‘people with data’.^42^ This means changing methods for engaging publics from techniques that monitor public attitudes to research over time to processes that realise the value of people’s health knowledges to support cross species translational research. Campbell suggests participating in these projects requires ‘a major and long-term commitment [which] must depend on both a strong motivation to assist in the project and a high level of trust’,^43^ over and above that required for other forms of scientific participation.^43,44^ Carefully targeted public engagement is essential for successful recruitment; socially acceptable ethical processes for consent, feedback and access, and agreement on how the biobank is used.^43,45^

Yet, there is a cross-over between engaging individuals as ‘people with data’ and the broader public interest. Biobanks construct publics as sample populations through appealing to notions of public good to solicit contributions and draw on local historical and political contexts to produce appropriate governance.^46^ The UK Biobank’s recruitment strategy involves appeals to ideas of ‘social solidarity embodied in the welfare state and the National Health Service’,^39^ invoking citizenship rather than personal health concerns to encourage and sustain participation. The people and patients contributing data and samples are important to realising future benefits from genome editing. Ongoing public trust in biobanks depends on many factors also important to public confidence in animal research including motivation, openness and governance.^47^ Many biobank–public interfaces are currently ‘simply unexplored’ or the data is ‘inconsistent’.^48^ We suggest more attention could be given to biobank participants as one of the publics assembled around genome editing and animal research to acknowledge their importance and explore how far biobank engagement depends on sustaining trust as rates of research, unrealistic expectations and commercial imperatives expand across related areas of science.

### Publics as participants

Other publics are more explicitly involved in shaping the priorities and practices of animal research. Patient and public involvement (PPI) is increasingly formalised within the funding, development and governance of health research^49^ and is recognised as a priority for genomics research.^50^ In PPI, publics are invited to co-produce all aspects of research, from setting research priorities to facilitating clinical trials. Early work by AIDS activists in the USA and the UK challenged researchers’ approaches to conducting trials that overlooked patients’ preferred outcomes.^51^ Further PPI has led to recognition of the ‘expert patient’ or ‘expert-by-experience’^52^ and patients are increasingly valued by researchers for their personal knowledge of their health conditions.^53^ Patient involvement can enable researchers to concentrate on the issues that matter to people,^54^ reduce research waste and build science that commands public trust.^55^ The Alzheimer’s Society, for example, suggest their patient involvement helped understand the ‘real world’ realities of dementia, enhance the validity of their methods, produce more useful and relevant outputs, and better navigate ethical issues and approvals.^56^ Advocacy groups also play an increasingly important role in collaborating with researchers, shaping the availability of research funding for things like rare diseases,^57^ to the extent that this public sociality can be seen as driving science.^58^

The abstract figure of ‘the patient’ has long been a powerful actor in arguments around animal research, but this has mostly involved them being ‘spoken for’ by advocates of animal research, rather than representing their own lived expertise.^59^ PPI repositions patients from health consumers to being ‘involved in all stages of research’,^51^ including in discussions about genome editing and animal research,^50^ though some PPI may be motivated by efforts to secure legitimacy or funding, rather than opening up decision-making. However, members of the public are likely to be actively involved in making future decisions about genome editing, influencing what research is funded and prioritised, as well as in the dissemination of results.^50^ PPI does not involve ‘lay’ stakeholders acquiring equivalent expertise to researchers, or speaking for all patients. Yet even in this specific context, the fact people are playing different roles is evidenced by the diverse terminologies used to define their involvement as ‘volunteers’, ‘consumers’ and ‘lay-members’ across different organisations.

Our own work has traced how people who are patients or carers involved in PPI manage their complex positions when reviewing and monitoring projects involving animal research.^60^ They may draw on personal or family health experiences, speak for patient communities, ask questions about animal care, or seek to offer public assurance around the regulation of animal research and welfare.^60^ The lines between public and personal interests become increasingly blurred. People draw on personal associations even when expressing ‘civic’ concern,^61^ and patients given personal access to animal research take their public responsibilities seriously.^59,60^ These expert patients are very different to the autonomous individual who is the imagined target of much public engagement or surveys, but they can make valuable contributions to the ‘knowledge engagement’ required to realise public benefits from genome editing using animals.^27^

## Discussion

The different publics and qualities of ‘publicness’^62^ outlined above are useful for considering why, how and when to involve publics in discussions about the application of genome editing to animal research. They help describe why diverse public views do not easily aggregate into a picture of what *the* public thinks about genome editing and explain how contradictory societal perspectives emerge from everyday experiences. They assist in identifying different ideas of public interest and the public good that underpin processes used to engage people. They also demand that those seeking to capture public views are clear about why they are doing this, how to do it, and how to use them. Efforts to measure public views change who makes up the public, what is the public interest, and how people respond.

However, this is not to promote only diversity or suggest public opinion surveys have no value. Following Dewey,^63^ we suggest well-crafted surveys can serve citizenship functions by creating discussion of what public opinion is or might be. A well-crafted survey would be explicit about how it is claiming to be representative of the perspectives around animal research, whether through prioritising demographic characteristics,^17^ patient views,^64^ or the range of positions on animal use,^65^ and reflexive about how these contribute to different notions of the public interest around animal research. We also argue that there are important commonalities in how people talk about genome technologies, which reflect how debates take place in the public domain. Some of this convergence results from the way public debates around science are mediated by similar research methodologies and media narratives, but it is also shaped by what people consistently value in their everyday lives, and their expectation that others will act in a capacity that is trustworthy. These insights can be used to nurture more positive knowledge exchange between patients, biobank participants, publics and scientists.

As the scope of genome editing expands, so does the diversity of issues and people it affects and involves, requiring new understandings of how to engage these emerging publics. We would follow Hartley et al. in calling for processes that maximise knowledge engagement across diverse publics, rather than simply public engagement.^27^ New forums are required to work with the diversity of publics, nationally and internationally, including all participants in reflexive exploration of ‘what questions should be asked, whose views must be heard, what imbalances of power should be made visible, and what diversity of views exist’.^66^ In taking approaches that reflect carefully, critically and contextually on their methods, efforts to understand public opinion can more constructively recognise how genomic editing is made meaningful and at times problematic in the everyday.

## References

[1] van Mil A Hopkins H Kinsella S. Potential uses for genetic technologies: Dialogue and engagement research conducted on behalf of the Royal Society. London: Royal Society.

[2] Nuffield Council on Bioethics. *Public dialogue on genome editing: Why? When? Who?* https://www.nuffieldbioethics.org/blog/public-dialogue-on-genome-editing-why-when-who (2016, accessed 26 August 2020).

[3] Irwin A. Constructing the scientific citizen: science and democracy in the biosciences. Public Underst Sci 2001; 10: 1–18.

[4] Irwin A. The politics of talk: coming to terms with the ‘new’ scientific governance. Soc Stud Sci 2006; 36: 299–320.

[5] Chilvers J Kearnes M. Remaking Participation: Science, environment and emergent publics. Abingdon: Routledge, 2015.

[6] Bruce A. Genome edited animals: learning from GM crops? Transgenic Res 2017; 26: 385–398. 28432545 10.1007/s11248-017-0017-2 PMC5422448

[7] Davies G Gorman R Greenhough B , et al. Animal research nexus: a new approach to the connections between science, health, and animal welfare. Med Humanit 2020; 46: 499–511. 32075866 10.1136/medhum-2019-011778 PMC7786151

[8] Hartley J. The Politics of Pictures: The creation of the public in the age of popular media. Hove: Psychology Press, 1992.

[9] Rawls J. Political Liberalism. New York: Columbia University Press, 1993.

[10] Ormandy EH Schuppli CA. Public attitudes toward animal research: a review. Animals 2014; 4: 391–408. 26480314 10.3390/ani4030391 PMC4494309

[11] Austin JL. How to do things with words. Oxford: Clarendon Press, 1962.

[12] Frewer LJ Howard C Shepherd R. Public concerns in the United Kingdom about general and specific applications of genetic engineering: risk, benefit, and ethics. Sci Technol Hum Values 1997; 22: 98–124. 10.1177/016224399702200105 11654686

[13] Davison A Barns I Schibeci R. Problematic publics: a critical review of surveys of public attitudes to biotechnology. Sci Technol Hum Values 1997; 22: 317–348.

[14] McNeil M Haran J. Publics of bioscience. Sci Cult(Lond) 2013; 22: 433–451.

[15] Gaskell G Allum N Bauer M , et al. Biotechnology and the European public. Nat Biotechnol 2000; 18: 935–938. 10973210 10.1038/79403

[16] European Commission. *Special Eurobarometer* *341: Biotechnology*, https://ec.europa.eu/commfrontoffice/publicopinion/archives/ebs/ebs_341_en.pdf (2010, accessed 26 August 2020).

[17] Ipsos MORI. *Attitudes to animal research: A long-term survey of public views 1999–2014*. A Report for the Department for Business Innovation & Skills.

[18] Understanding Animal Research. Concordat on Openness on Animal Research in the UK, http://concordatopenness.org.uk/ (2014, accessed 26 August 2020).

[19] Ipsos MORI. *Public attitudes to animal research in 2018*, https://www.ipsos.com/ipsos-mori/en-uk/public-attitudes-animal-research-2018 (accessed 26 August 2020).

[20] Hobson-West P. The role of ‘public opinion’ in the UK animal research debate. J Med Ethics 2010; 36: 46–49. 20026693 10.1136/jme.2009.030817 PMC5027911

[21] Smith RDJ Samuel G. *Who’s talking about non-human Genome Editing? Mapping public discussion in the UK.* Sciencewise. https://www.publicengagement.ac.uk/sites/default/files/publication/genome_editing_for_human_health_event_report_final_002.pdf (2018, accessed 5 January 2020)

[22] Robins R. Inventing Oncomice: making natural animal, research tool and invention cohere. Genom Soc Policy 2008; 4: 21–35.

[23] Brown N. Organising/disorganising the breakthrough motif: Dolly the cloned ewe meets Astrid the hybrid pig. In: Brown N Rappert B Webster A (eds) Contested Futures: A sociology of prospective techno-science. Abingdon: Routledge, 2000, pp. 87–108.

[24] Michael M Brown N. Scientific citizenships: self-representations of xenotransplantation’s publics. Sci Cult (Lond) 2005; 14: 39–57. 16178113 10.1080/09505430500041769

[25] Davies G. The sacred and the profane: biotechnology, rationality, and public debate. Environ Plan A 2006; 38: 423–443.

[26] Haran J. The UK hybrid embryo controversy: delegitimising counterpublics. Sci Cult (Lond) 2013; 22: 567–588.

[27] Hartley S Thizy D Ledingham K , et al. Knowledge engagement in gene drive research for malaria control. PLOS Negl Trop Dis 2019; 13: e0007233. 31022169 10.1371/journal.pntd.0007233 PMC6483158

[28] Macnaghten P. Animals in their nature: a case study on public attitudes to animals, genetic modification and ‘nature’. Sociology 2004; 38: 533–551.

[29] Marris C. Public views on GMOs: deconstructing the myths. EMBO Rep 2001; 2: 545–548. 11463731 10.1093/embo-reports/kve142 PMC1083956

[30] Rollin BE. The Frankenstein Syndrome: Ethical and social issues in the genetic engineering of animals. Cambridge: Cambridge University Press, 1995.

[31] Milne R. Pharmaceutical prospects: biopharming and the geography of technological expectations. Soc Stud Sci 2012; 42: 290–306. 22849000 10.1177/0306312711436266

[32] Sciencewise. *Genome Editing for Human Health: Report of a roundtable to explore future public engagement priorities*. Department for Business, Energy & Industrial Strategy, https://www.publicengagement.ac.uk/sites/default/files/publication/genome_editing_for_human_health_event_report_final_002.pdf (2018, accessed 26 August 2020).

[33] Department for Business, Energy & Industrial Strategy (BEIS). *Public attitudes to science 2019*. BEIS Research Paper Number 2020/012.

[34] Irwin A Wynne B. Misunderstanding Science? The public reconstruction of science and technology. Cambridge: Cambridge University Press, 2003.

[35] Wehling P. From invited to uninvited participation (and back?): rethinking civil society engagement in technology assessment and development. Poiesis Prax 2012; 9: 43–60. 23204995 10.1007/s10202-012-0125-2 PMC3510401

[36] Mulvihill JJ Capps B Joly Y , et al. Ethical issues of CRISPR technology and gene editing through the lens of solidarity. Br Med Bull 2017; 122: 17–29. 28334154 10.1093/bmb/ldx002

[37] Prainsack B Buyx A. Solidarity: Reflections on an emerging concept in bioethics. London: Nuffield Council on Bioethics, 2011.

[38] Samuel GN Farsides B. Public trust and ‘ethics review’ as a commodity: the case of Genomics England Limited and the UK’s 100,000 genomes project. Med Health Care Philos 2018; 21: 159–168. 29086191 10.1007/s11019-017-9810-1 PMC5884416

[39] Busby H Martin P. Biobanks, national identity and imagined communities: the case of UK biobank. Sci Cult 2006; 15: 237–251.

[40] Brown SDM Holmes CC Mallon A-M , et al. High-throughput mouse phenomics for characterizing mammalian gene function. Nat Rev Genet 2018; 19: 357–370. 29626206 10.1038/s41576-018-0005-2 PMC6582361

[41] Neuner SM Heuer SE Huentelman MJ , et al. Harnessing genetic complexity to enhance translatability of alzheimer’s disease mouse models: a path toward precision medicine. Neuron 2019; 101: 399–411.e5. 30595332 10.1016/j.neuron.2018.11.040 PMC6886697

[42] Milne R. From people with dementia to people with data: participation and value in Alzheimer’s disease research. BioSocieties 2018; 13: 623–639.

[43] Campbell AV. The ethical challenges of genetic databases: safeguarding altruism and trust. Kings Law J 2007; 18: 227–245.

[44] Critchley C Nicol D McWhirter R. Identifying public expectations of genetic biobanks. Public Underst Sci 2017; 26: 671–687. 26769748 10.1177/0963662515623925

[45] Haga SB Beskow LM. Ethical, legal, and social implications of biobanks for genetics research. Adv Genet 2008; 60: 505–544. 18358331 10.1016/S0065-2660(07)00418-X

[46] Tupasela A Snell K Cañada JA. Constructing populations in biobanking. Life Sci Soc Policy 2015; 11: 5. 26194269 10.1186/s40504-015-0024-0 PMC4508277

[47] Levitt DM Weldon S. A well placed trust? Public perceptions of the governance of DNA databases. Crit Public Health 2005; 15: 311–321.

[48] Gottweis H Chen H Starkbaum J. Biobanks and the phantom public. Hum Genet 2011; 130: 433–440. 21773770 10.1007/s00439-011-1065-y

[49] Staniszewska S Denegri S. Patient and public involvement in research: future challenges. Evid Based Nurs 2013; 16: 69–69. 23749800 10.1136/eb-2013-101406

[50] Nunn JS Tiller J Fransquet P , et al. Public involvement in global genomics research: a scoping review. *Front Public Health*; 7. Epub ahead of print 9 April 2019. DOI: 10.3389/fpubh.2019.00079. 10.3389/fpubh.2019.00079 PMC6467093 31024880

[51] Thornton H. Patient and public involvement in clinical trials. BMJ 2008; 336: 903–904. 18436920 10.1136/bmj.39547.586100.80 PMC2335270

[52] Noorani T. Service user involvement, authority and the ‘expert-by-experience’ in mental health. J Polit Power 2013; 6: 49–68.

[53] Caron-Flinterman JF Broerse JEW Bunders JFG. The experiential knowledge of patients: a new resource for biomedical research? Soc Sci Med 2005; 60: 2575–2584. 15814182 10.1016/j.socscimed.2004.11.023

[54] van der Scheer L Garcia E van der Laan AL , et al. The benefits of patient involvement for translational research. Health Care Anal 2017; 25: 225–241. 25537464 10.1007/s10728-014-0289-0

[55] Chalmers I Bracken MB Djulbegovic B , et al. How to increase value and reduce waste when research priorities are set. Lancet 2014; 383: 156–165. 24411644 10.1016/S0140-6736(13)62229-1

[56] Morgan N Grinbergs-Saull A Murray M. *‘We can make our research meaningful’: The impact of the Alzheimer’s Society Research Network*, https://www.alzheimers.org.uk/sites/default/files/2018-04/Research%20Network%20Report%20low-res.pdf (2018, accessed 2 November 2020).

[57] Dunkle M Pines W Saltonstall PL. Advocacy groups and their role in rare diseases research. In: Posada de la Paz M Groft SC (eds) Rare diseases epidemiology. Dordrecht: Springer Netherlands, pp. 515–525. 10.1007/978-90-481-9485-8_28 20824463

[58] Panofsky A. Generating sociability to drive science: patient advocacy organizations and genetics research. Soc Stud Sci 2011; 41: 31–57. 21553639 10.1177/0306312710385852

[59] Davies G Gorman R Crudgington B. Which patient takes centre stage? Placing patient voices in animal research. In: Atkinson S Hunt R (eds) GeoHumanities and health. Cham: Springer, 2020, pp. 141–155. 31829542

[60] Gorman R Davies G. When ‘cultures of care’ meet: entanglements and accountabilities at the intersection of animal research and patient involvement in the UK. *Soc Cult Geogr*. Epub ahead of print 3 September 2020. DOI: 10.1080/14649365.2020.1814850 10.1080/14649365.2020.1814850 PMC9872947 36712288

[61] Marres N. The issues deserve more credit: pragmatist contributions to the study of public involvement in controversy. Soc Stud Sci 2007; 37: 759–780.

[62] Newman J Clarke J. Publics, politics and power: Remaking the public in public services. London: SAGE, 2009.

[63] Dewey J. The public and its problems. New York: Holt, 1927.

[64] Masterton M Renberg T Sporrong SK. Patients’ attitudes towards animal testing: ‘To conduct research on animals is, I suppose, a necessary evil’. BioSocieties 2014; 9: 24–41.

[65] Schuppli CA Weary DM. Attitudes towards the use of genetically modified animals in research. Public Underst Sci 2010; 19: 686–697. 21560543 10.1177/0963662510362834

[66] Jasanoff S Hurlbut JB. A global observatory for gene editing. Nature 2018; 555: 435–437. 10.1038/d41586-018-03270-w 29565415

